# Characterization of Genomic Vitamin D Receptor Binding Sites through Chromatin Looping and Opening

**DOI:** 10.1371/journal.pone.0096184

**Published:** 2014-04-24

**Authors:** Sabine Seuter, Antonio Neme, Carsten Carlberg

**Affiliations:** School of Medicine, Institute of Biomedicine, University of Eastern Finland, Kuopio, Finland; University of Oxford, United Kingdom

## Abstract

The vitamin D receptor (VDR) is a transcription factor that mediates the genomic effects of 1α,25-dihydroxyvitamin D_3_ (1,25(OH)_2_D_3_). Genome-wide there are several thousand binding sites and hundreds of primary 1,25(OH)_2_D_3_ target genes, but their functional relation is largely elusive. In this study, we used ChIA-PET data of the transcription factor CTCF in combination with VDR ChIP-seq data, in order to map chromatin domains containing VDR binding sites. In total, we found 1,599 such VDR containing chromatin domains and studied in THP-1 human monocytic leukemia cells four representatives of them. Our combined ChIP-seq and FAIRE-seq time course data showed that each of these four domains contained a master VDR binding site, where an increase of VDR binding pairs with 1,25(OH)_2_D_3_-promoted chromatin opening and the presence of a highly significant DR3-type sequence below the peak summit. These sites differed in their relative VDR binding but not in their kinetics, while other loci either had a weaker and delayed VDR association or could not be confirmed at all. All studied chromatin domains contained at least one primary 1,25(OH)_2_D_3_ target gene demonstrating a characteristic slope of mRNA increase, while neighboring genes responded delayed, if at all. In conclusion, the observation of ligand-inducible VDR binding and chromatin opening combined with a DR3-type sequence highlighted genome-wide 160 VDR loci that have within their chromatin domain a more than 4-fold increased likelihood to identify a primary 1,25(OH)_2_D_3_ target gene than in the vicinity of other genomic VDR binding sites.

## Introduction

The nuclear receptor VDR belongs to a unique transcription factor superfamily, whose members are directly activated by small lipophilic compounds [1]. Accordingly, the biologically most active vitamin D compound, 1,25(OH)_2_D_3_, is the specific high-affinity ligand of VDR [2]. Active vitamin D regulates calcium and phosphate homeostasis and therefore has a major impact on bone mineralization [3], but 1,25(OH)_2_D_3_ also has cell growth-related and immunomodulatory functions [4], [5]. Hematopoietic cells, such as monocytes and macrophages, are important targets of 1,25(OH)_2_D_3_ [6]. The human monocytic leukemia cell line THP-1 has been used previously [7]–[10] for investigations of the effects of 1,25(OH)_2_D_3_ in the context of immunity and cellular proliferation. Primary VDR target genes are identified by short incubations (2 to 4 h) with 1,25(OH)_2_D_3_, but the physiological impact of the receptor and its ligand gets more obvious after stimulations for 24 h or longer. In THP-1 cells, transcriptome analyses indicated that already 408 genes are significantly up-regulated after 4 h stimulation with 1,25(OH)_2_D_3_ [7], while after 24 h, even 1,651 genes showed increased expression [11].

VDR binds preferentially to sequences, which are direct repeats of two hexameric binding sites with three spacing nucleotides (DR3) [12], [13]. However, the intrinsic repressive nature of chromatin denies VDR the access to many putative binding sites on genomic DNA [14], [15]. Genomic regions with open chromatin can be detected genome-wide by the method Formaldehyde-Assisted Isolation of Regulatory Elements sequencing (FAIRE-seq), which identifies chromatin sites depleted of nucleosomes [16]–[18]. VDR binds genomic DNA already in the absence of ligand, when it forms complexes with co-repressor proteins and histone deacetylases [19], [20]. In contrast, in the presence of 1,25(OH)_2_D_3_ VDR associates with co-activator proteins and histone acetyltransferases [21]. The control of transcription involves the formation of physical connections between transcription start sites (TSSs) and transcription factor binding sites [22]. Therefore, a VDR binding site should be located within the same chromatin domain as the gene(s) that it is controlling. In this way, mediator proteins are able to link ligand-activated VDR to the basal transcriptional machinery [23] resulting in transcriptional activation [14].

The method chromatin immunoprecipitation sequencing (ChIP-seq) allows the monitoring of all genome-wide binding sites of transcription factors [24]. So far, VDR ChIP-seq data had been published from five different human cellular models: the lymphoblastoid cells GM10855 and GM10861 [25], the monocyte-like cells THP-1 [7], LS180 colon cancer cells [26] and LX2 hepatic stellate cells [27]. These studies reported 1,600–6,200 VDR-specific binding sites. In addition, the first study of genome-wide VDR binding in primary CD4^+^ T-lymphocytes, which were obtained from nine healthy human volunteers, reported between a few hundred and more than 10,000 genome-wide VDR binding sites [28]. Taken together, the ChIP-seq studies suggest that in most cellular systems there are far more genomic VDR binding sites than target genes. For only a minority of these VDR loci a target gene has been assigned, i.e. the function of most of these sites is still elusive.

Chromatin loops occur when genomic sequences from the same chromosome are in close physical proximity to each other [29], e.g. at insulator regions. The evolutionarily highly conserved protein CCCTC-binding factor (CTCF) is a central insulator binding factor [30], i.e. it is often found in genomic regions that separate genomic domains from each other [31]. The method chromatin interaction analysis by paired-end tag sequencing (ChIA-PET) [32] maps the interaction between protein-associated genomic regions. When applied for CTCF in K562 human monocytic leukemia cells [33], it indicated 120,000 intra-chromosomal, CTCF-mediated chromatin interactions representing differently sized chromatin domains. In this study, we aimed to extrapolate the CTCF ChIA-PET data from K562 cells to the closely related THP-1 cells, in order to determine in combination with our VDR ChIP-seq data VDR-containing chromatin domains. We characterized four representative VDR binding sites by ligand-dependent VDR binding and chromatin opening and assigned them to primary 1,25(OH)_2_D_3_ target genes. This should allow a segregation of master VDR loci from less important sites and a more efficient identification and characterization of 1,25(OH)_2_D_3_ target genes.

## Materials and Methods

### Cell Culture

THP-1 cells [34] was grown in RPMI 1640 medium supplemented with 10% fetal calf serum, 2 mM L-glutamine, 0.1 mg/ml streptomycin and 100 U/ml penicillin and the cells were kept at 37°C in a humidified 95% air/5% CO_2_ incubator. Prior to chromatin or mRNA extraction, cells were grown overnight in phenol red-free medium supplemented with charcoal-stripped fetal calf serum. Then, cells were treated with solvent (0.1% ethanol) or 100 nM 1,25(OH)_2_D_3_ (Sigma-Aldrich) for the indicated time periods.

### ChIP

ChIP was performed exactly as reported before [35]. Selected genomic regions containing VDR peaks were analyzed by quantitative polymerase chain reaction (qPCR) using equal DNA amounts of chromatin fragments, a SYBRGreen I master mix (Roche) and the specific primer pairs (Table S1). The qPCR reactions were performed using the following profile: 10 min at 95°C, followed by 45 cycles of 20 s at 95°C, 15 s at primer-specific annealing temperature (Table S1) and 15 s at 72°C and a final amplification step of 10 min at 72°C. The results were normalized with respect to input by using the formula 2^−(ΔCt)^*100, where ΔCt is Ct_(input)_ – Ct_(immunoprecipitated DNA)_ and Ct is the fractional cycle number.

### ChIP-seq, FAIRE-seq and ChIA-PET Data Analysis and Visualization

Publically available CTCF ChIP-seq datasets of the ENCODE consortium [33] were downloaded for K562 human monocytic leukemia cells (wgEncodeEH002279) and MCF-7 human breast carcinoma cells (wgEncodeEH001132) using the UCSC genome browser (http://genome.ucsc.edu/ENCODE). Our own VDR ChIP-seq (GSE27437) and FAIRE-seq (GSE40075) datasets are available at GEO (www.ncbi.nlm.nih.gov/geo). ChIP-seq and FAIRE-seq data were visualized by using the Integrative Genomics Viewer (IGV) [36]. CTCF ChIA-PET data from K562 cells (wgEncodeEH002075) and MCF-7 cells (wgEncodeEH002076) were visualized using the UCSC genome browser (http://genome.ucsc.edu) [37]. The size of VDR containing chromatin loops was determined by counting the number of bases separating the summit of the nearest CTCF peak to the left and the summit of the nearest CTCF peak to the right of the VDR binding sites [7]. If no CTCF peak is found between the peak and the start or end of the chromosome, the respective chromosome end points are considered as the natural limit of the domain. The software tool HOMER [38] with a minimal score of 7 identified DR3-type sequences below VDR peak summits (+/−100 bp).

### qPCR

Total RNA extraction and cDNA synthesis was done as reported before [35]. qPCR reactions were performed using 250 nM of reverse and forward primers (Table S2), 2 µl 1/20 diluted cDNA template and the LightCycler 480 SYBRGreen I Master mix (Roche) in a total volume of 10 µl. In the PCR reaction, the hotstart Taq polymerase was activated for 10 min at 95°C, followed by 40 amplification cycles of 20 s denaturation at 95°C, 15 s annealing at primer-specific temperatures (Table S2) and 15 s elongation at 72°C and a final elongation for 10 min at 72°C. PCR product specificity was monitored using post-PCR melt curve analysis. Relative expression levels were determined with the comparative delta threshold cycle (ΔCt) method. Relative expression levels of the target genes were normalized to the three most stable out of ten tested internal reference genes (*B2M*, *GAPDH* and *HPRT1*). The stability of the expression of the reference genes was determined using the geNorm algorithm [39]. Briefly, the arithmetic mean of replicated Ct values for each gene is transformed to a relative quantity (setting the sample with the highest expression as calibrator to 1), using the ΔCt formula Q  = 2^ΔCt^  =  2^(calibratorCt – sampleCt)^ (Q  =  quantity sample relative to the calibrator sample). For the normalization, the relative quantities were divided by the normalization factor being the geometric mean of the three reference genes.

## Results

### VDR Binding Sites within Chromosomal Domains

**Figure 1:**
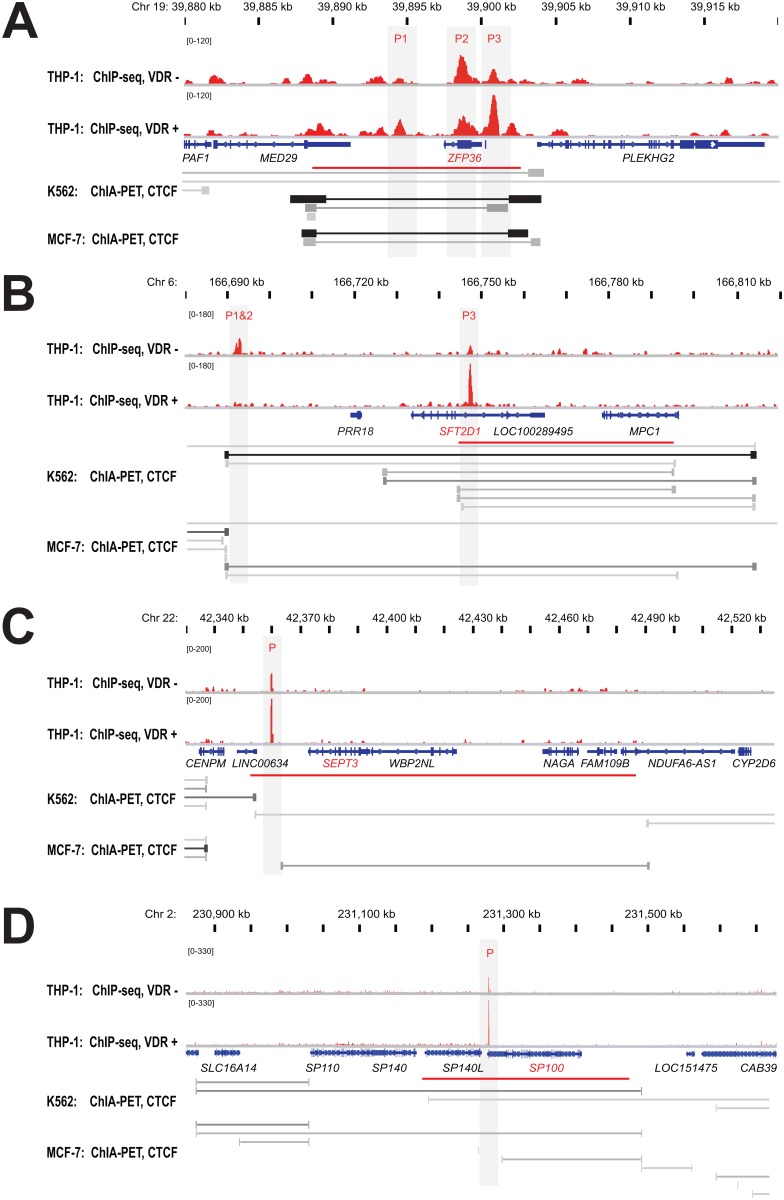
Chromatin loops containing VDR binding sites. The IGV browser was used to display the chromatin loops around the genes *ZFP36* (A), *SFT2D1* (B), *SEPT3* (C) and *SP100* (D). VDR ChIP-seq data from THP-1 cells [7] (unstimulated (−) and treated for 40 min with 1,25(OH)_2_D_3_ (+), red) are shown in comparison with CTCF ChIA-PET data from K562 and MCF-7 cells [33] in the looping view (grey horizontal lines). Horizontal red lines indicate the core chromatin loops (as indicated in Table S3). The area of the genomic regions was adapted to the size of the chromatin loops. Gene structures are shown in blue, and VDR peak regions are shaded in grey.

In order to describe genome-wide chromatin loops, we used ENCODE data of the 3-dimensional interactions of CTCF as determined by ChIA-PET assays performed in K562 cells [33]. The human monocytic leukemia cell lines K562 [40] and THP-1 [34] are far closer to each other than the breast cancer cell line MCF-7 [41]. However, CTCF ChIP-seq data as well as CTCF ChIP-PET data from even K562 and MCF-7 cells are very similar to each other (Fig. S1). This allowed us to assume that the information on chromatin domains from K562 cells can be extrapolated to THP-1 cells. We defined core chromatin domains containing VDR binding sites (red horizontal lines in Fig. 1) by determining the distance between the closest ChIA-PET CTCF peaks left and right of the 2,340 VDR ChIP-seq peaks in THP-1 cells [7]. This identified 1,599 chromatin domains containing one or more VDR binding sites (Table S3). The size of these chromatin domains ranges from less than 1 kb to up to 21 Mb (Fig. S2). When dividing the list of domains into quartiles, the respective average sizes are 12.5, 51.5, 124 and 596 kb, respectively.

For each of the four quartiles, we selected the following representative genomic regions: i) a short chromatin domain of 12.5 kb containing three VDR binding sites close to the ZFP36 ring finger protein (*ZFP36*) gene (Fig. 1A), ii) a domain of 50 kb including one VDR site (and two additional sites, when considering a larger loop, see below) around the SFT2 domain containing 1 (*SFT2D1*) gene (Fig. 1B), iii) a mid-sized domain of 132 kb with one VDR site close to the septin 3 (*SEPT3*) gene (Fig. 1C) and iv) a very large domain of 288 kb with one VDR site next to the SP100 nuclear antigen (*SP100*) gene (Fig. 1D). We have chosen these chromatin domains, because their respective major VDR peak is located closest to the TSS of the lead primary 1,25(OH)_2_D_3_ target genes in the genomic region, as indicated by microarray data in the same cellular model [7]. However, the chosen chromatin domains contain two to six additional genes, which may be all VDR targets. In addition to the core chromatin domains, the ChIA-PET data also suggest a number of larger loops defined by more distant CTCF sites. Therefore, we adjusted the scales of Figs. 1A–D, in order to display loops in the size of 17, 125, 177 and 590 kb around the genes *ZFP36*, *SFT2D1*, *SEPT3* and *SP100*, respectively. For the continuation of this study we refer to these extended chromatin domains.

In summary, 3-dimensional CTCF-mediated looping data allowed a segregation of the human genome into chromatin loops, some 1,600 of which include at least one VDR binding site. The size of these chromatin domains varies significantly. Four genomic regions (17 to 590 kb) representing this size range indicated that the number of contained VDR sites does not depend upon the loop size.

### Chromatin Accessibility at VDR Binding Sites

**Figure 2:**
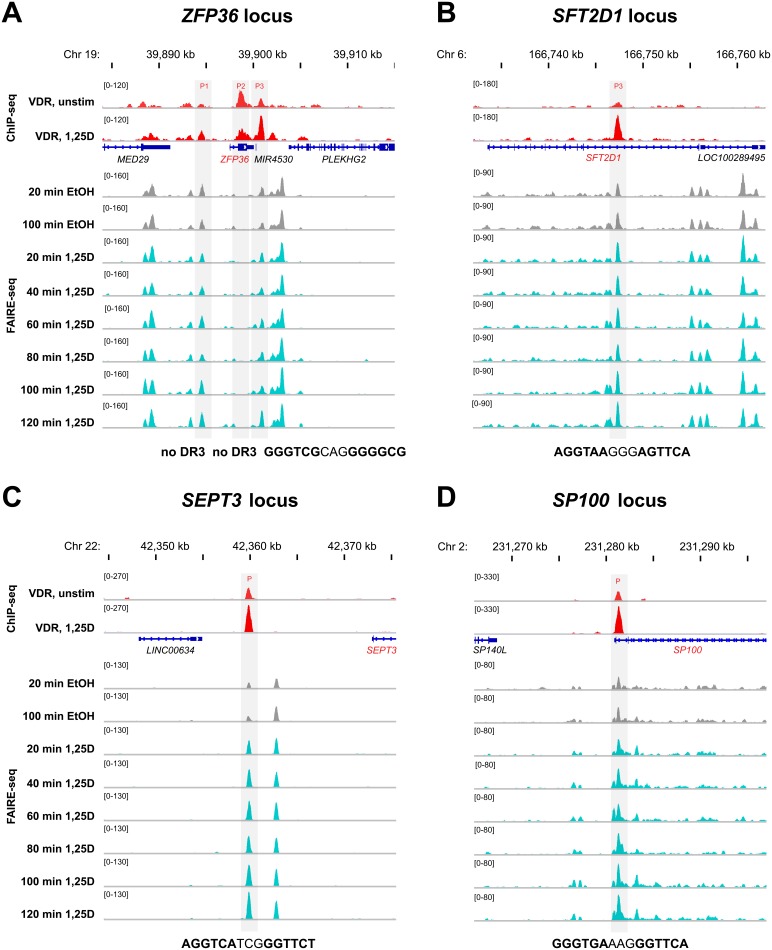
VDR association and 1,25(OH)_2_D_3_-dependent chromatin opening in four chromatin domains. The IGV browser was used to visualize the loci of the genomic VDR binding regions (+/−15 kb of the peak summit) of the genes *ZFP36* (A), *SFT2D1* (B), *SEPT3* (C) and *SP100* (D). The peak tracks display data from a VDR ChIP-seq experiment in THP-1 cells (red, from unstimulated cells and after 40 min 1,25(OH)_2_D_3_ (1,25D) treatment [7]) and a time course of FAIRE-seq data from THP-1 cells (grey for EtOH-treated controls and turquoise for 1,25(OH)_2_D_3_ treatments for the indicated time periods [35]). Gene structures are shown in blue, and VDR peak regions are shaded in grey. In the bottom lines, the sequences of DR3-type binding sites are indicated.

For a more detailed characterization of the eight VDR binding sites within the four representative genome domains, we described the relation to chromatin accessibility and 1,25(OH)_2_D_3_-induced chromatin opening with the help of FAIRE-seq time course data [35] from THP-1 cells (Fig. 2). From the three VDR binding sites within the chromatin domain of the *ZFP36* gene, only the first (P1*_ZFP36_*, 3.0 kb upstream of the *ZFP36* TSS) and the third (P3*_ZFP36_*, 3.4 kb downstream of the *ZFP36* TSS) were associated with open chromatin. In contrast, at the site of the second VDR peak (P2*_ZFP36_*, 1.2 kb downstream of the *ZFP36* TSS) no sign of chromatin accessibility could be detected (Fig. 2A). Interestingly, at P2*_ZFP36_* ChIP-seq data suggested decreased VDR binding after stimulation with ligand, while at P1*_ZFP36_* and P3*_ZFP36_* a clear ligand-dependent increase of VDR association could be detected. Furthermore, at P1*_ZFP36_* and P3*_ZFP36_* also a slight increase (1.48- and 1.43-fold, Table S3) of the rate of chromatin opening was observed. However, only below the summit (+/−100 bp) of P3*_ZFP36_* a DR3-type binding sequence with a highly significant HOMER score of 8 could be detected but not below the summits of P1*_ZFP36_* and P2*_ZFP36_*. From the three VDR peaks within the maximal chromatin loop of the *SFT2D1* gene, P1*_SFT2D1_* and P2*_SFT2D1_* were closely located to each other 29 and 28 kb upstream of the gene’s TSS (Fig. S3). In our ChIP-seq analysis [7], the VDR binding to both sites was not considered to be statistically significant, i.e. they were not part of the dataset of 2,340 reported VDR peaks, but for reference we included them in this study. Both sites do not contain a DR3-type sequence. Site P1*_SFT2D1_* was constitutively associated with open chromatin, while at site P2*_SFT2D1_* no sign of accessible chromatin could be detected. In contrast, the third VDR binding site within the *SFT2D1* chromatin domain (P3*_SFT2D1_*, 8.5 kb downstream of the TSS, Fig. 2B) as well as the single VDR binding sites of the *SEPT3* domain (P*_SEPT3_*, 12 kb upstream of the TSS, Fig. 2C) and the *SP100* domain (P*_SP100_,* 0.5 kb downstream of the TSS, Fig. 2D) showed the same profile. At all three sites VDR binding and chromatin accessibility were prominently increased by ligand (with the exception of the chromatin opening at P*_SP100_*, which is only a non-significant 1.14-fold induction). Moreover, below each of the peak summits a DR3-type binding sequence is located.

The four exemplified master VDR binding sites belong to a group of 160 (6.8%) VDR peaks sharing these properties (Table S3 and Fig. S4). Our criteria to assign a VDR peak to the list of master sites were i) a VDR ChIP-seq fold enrichment of more than 9 (applying to 627 of the 2,340 peaks), ii) a FAIRE-seq signal that is more than 1.1-fold increased after ligand stimulation (709 peaks) and iii) carrying a DR3-type sequence below the VDR peak summit (+/−100 bp) with a HOMER score of 7 and higher (739 peaks). Interestingly, all these sites occur in isolation, i.e. there is only one master VDR binding site per loop. From the 408 genes that are significantly up-regulated after a 4 h induction with 1,25(OH)_2_D_3_ [7], 179 (43.9%) are located within the 1,599 VDR containing chromatin domains (Table S4). From these 179 domains 52 (29.1%) enclose a master VDR site whereas only 6.8% of all 2,340 VDR binding sites are master VDR loci.

Taken together, FAIRE-seq time course data were well suited for a detailed analysis of VDR binding sites in relation to chromatin accessibility. Each of the four investigated chromatin loops carried only one site, where an increase of VDR binding was associated with 1,25(OH)_2_D_3_-promoted chromatin opening and the presence of a high confidence DR3-type binding sequence. In total, there are 160 chromatin loops with a single master VDR site in THP-1 cells. The presence of a master VDR site increases the likelihood to identify a primary 1,25(OH)_2_D_3_ target gene in the same chromatin domain by a factor of more than 4.

### 1,25(OH)_2_D_3_-dependent VDR Association

**Figure 3:**
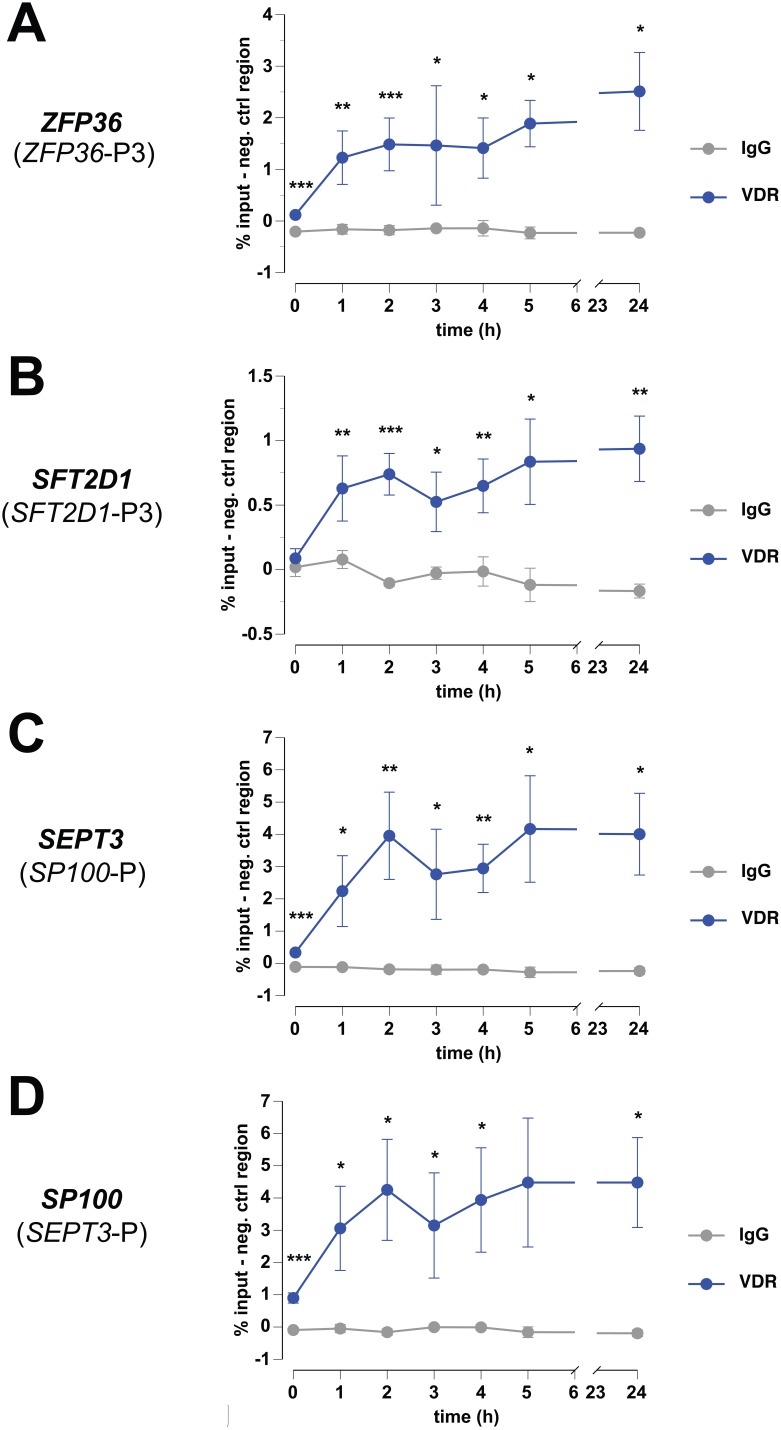
Dynamics of VDR association. ChIP-qPCR was performed to determine VDR association (blue) and unspecific IgG binding (grey) at P3*_ZFP36_* (A), P3*_SFT2D1_* (B), P*_SEPT3_* (C) and P*_SP100_* (D). THP-1 cells were stimulated for 1, 2, 3, 4, 5 and 24 h with 100 nM 1,25(OH)_2_D_3_ and chromatin was extracted. The data points represent the means of at least three independent experiments and the bars indicate standard deviations. Unspecific background binding observed to a negative control region of the *MB* gene (see Fig. S5E) was subtracted. Two-tailed Student’s t-tests were performed to determine the significance of VDR association in reference to IgG background (*p<0.05; **p<0.01; ***p<0.001).

The observation that at some VDR binding sites chromatin opens after stimulation with 1,25(OH)_2_D_3_ (Fig. 2) led to the question, whether VDR associates with these sites also in a time-dependent fashion. Therefore, we performed at the master VDR sites of the four representative genomic regions ChIP time courses with measurements 1, 2, 3, 4, 5, and 24 h after stimulation with 1,25(OH)_2_D_3_ (Fig. 3), i.e. far longer time periods than in our previous studies [35], [42]. These ChIP-qPCR experiments could confirm VDR binding to the four sites and suggested for all of them very similar binding kinetics: a rather rapid association of VDR within 1 h reached after approximately 2 h saturation, i.e. VDR binding at 2 and 24 h did not differ significantly. However, the saturation levels of VDR binding seemed to be site-specific: for P3*_ZFP36_* there was some 2% binding compared to the input reference (Fig. 3A), below 1% for P3*_SFT2D1_* (Fig. 3B), some 4% for P*_SEPT3_* (Fig. 3C) and nearly 5% for P*_SP100_* (Fig. 3D).

For comparison, we investigated in the same way the time-dependent VDR binding at P1*_ZFP36_*, P2*_ZFP36_*, P1*_SFT2D1_* and P2*_SFT2D1_* (Fig. S5). At P1*_ZFP36_*, we observed a slow and weak but significant up-load of VDR reaching a saturation level of less than 0.5% (Fig. S5A). In contrast, neither at P2*_ZFP36_* (Fig. S5B) nor at P1*_SFT2D1_* (Fig. S5C) or P2*_SFT2D1_* (Fig. S5D) we found any significant VDR association. At these three sites, VDR did not differ from background binding to the negative control region (exon 2 of the myoglobin (*MB*) gene, Fig. S5E) nor from the association with unspecific IgG. This means that the three latter sites could not be validated as VDR binding loci, which concerning P1*_SFT2D1_* and P2*_SFT2D1_* agreed with our ChIP-seq data analysis [7].

In summary, the master VDR binding sites of the investigated chromatin loops could be confirmed by ChIP-qPCR. They differed in their relative receptor binding extent but not in the kinetics of VDR binding. The additional VDR binding sites contained in these four chromatin domains either had a much weaker and delayed VDR association or could not be confirmed at all.

### Functional Consequences on mRNA Expression

Next, we tested, whether the binding of VDR and the 1,25(OH)_2_D_3_-induced chromatin opening have any functional consequences for the mRNA expression of the genes within the four representative chromatin loops. First, we monitored the basal expression of all genes, which have their TSS region within the tested chromatin regions (Fig. S6). In its small chromatin loop, the *ZFP36* gene showed equal basal expression as the *MED29* (mediator complex subunit 29) gene and nearly 9-times higher mRNA amounts than the *PLEKHG2* (pleckstrin homology domain containing, family G member 2) gene (Fig. S6A). The *SFT2D1* gene displayed highest basal expression within its chromatin loop and is 1.8-, 59- and 652-times higher expressed than its neighboring genes *MPC1* (mitochondrial pyruvate carrier 1), *PRR18* (proline rich 18) and the uncharacterized *LOC100289495*, respectively (Fig. S6B). The basal mRNA levels of the *SEPT3* gene is 36-, 14-, 11- and 7-times lower than those of the surrounding genes *NDUFA6* (NADH dehydrogenase (ubiquinone) 1 alpha subcomplex 6), *SMDT1* (single-pass membrane protein with aspartate-rich tail 1), *NAGA* (N-acetylgalactosaminidase, alpha) and *CENPM* (centromere protein M), respectively, but 10- and 23-times higher than those of the genes *WBP2NL* (WBP2 N-terminal like) and *FAM109B* (family with sequence similarity 109, member B) (Fig. S6C). The basal expression of the *SP100* gene is 17-times lower than that of the *CAB39* (calcium binding protein 39) gene, nearly equal to that of the genes *SP110* (SP110 nuclear body protein) and *SP140L* (SP140 nuclear body protein-like), 5- and 18- times higher than that of the genes *SP140* (SP140 nuclear body protein) and *SLC16A14* (solute carrier family 16, member 14) (Fig. S6D).

**Figure 4:**
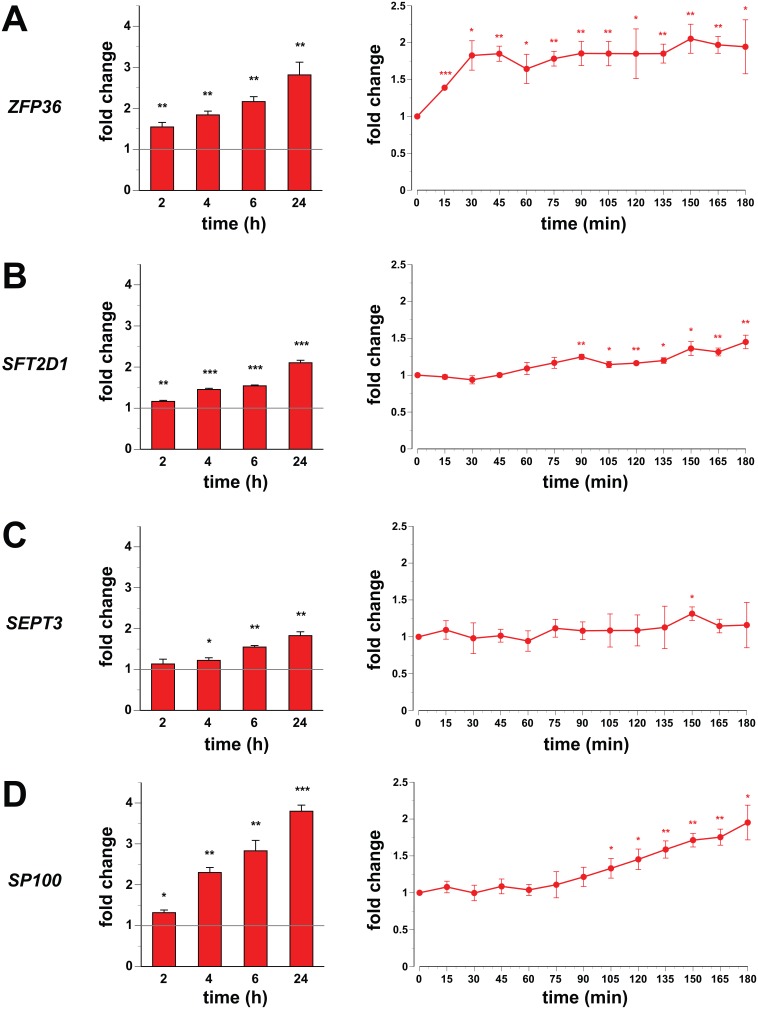
Detailed time course expression profiling of primary VDR target genes. qPCR was performed to determine the relative changes of mRNA expression of the genes *ZFP36* (A), *SFT2D1* (B), *SEPT3* (C) and *SP100* (D) normalized by the three reference genes *B2M*, *GAPDH* and *HPRT1*. THP-1 cells were incubated with 100 nM 1,25(OH)_2_D_3_ for either 2, 4, 6 and 24 h (left) or at 15 min intervals over a time period of 180 min (right). The columns (left) or data points (right) represent the means of three independent experiments (each performed in triplicate) and the bars indicate standard deviations. Two-tailed Student’s t-tests were performed to determine the significance of the mRNA induction by 1,25(OH)_2_D_3_ in reference to solvent-treated cells (*p<0.05; **p<0.01; ***p<0.001).

All 20 genes within the four chromatin domains were tested for the effects of 1,25(OH)_2_D_3_ on their expression 2, 4, 6 and 24 h after onset of stimulation (Figs. 4 (left) and S7). Only the genes *ZFP36* (Fig. 4A), *SFT2D1* (Fig. 4B), *SEPT3* (Fig. 4C) and *SP100* (Fig. 4D) displayed a consistent up-regulation, which was already after 2 h (*ZFP36*, *SFT2D1* and *SP100*) or at least after 4 h (*SEPT3*) statistically significant. The maximal induction of their mRNA accumulation varied between 1.8- (*SEPT3*) and 3.8-fold (*SP100*). The *ZPF36* flanking gene *PLEKHG2* showed a 1.8-fold induction after 6 h ligand stimulation (Fig. S7A), the *SFT2D1* neighbor *MPC1* increased 2.3-fold after 24 h (Fig. S7B), the *SEPT3* loop member *NDUFA6* displayed a 1.7-fold higher mRNA level after 24 h (Fig. S7C) and the *SP100* flanking gene *SP140L* rose 1.4-fold after 24 h (Fig. S7D). The mRNA of the remaining 12 genes either did not change statistically significantly or their induction was below 1.3-fold (Fig. S7).

Finally, we were interested in the short-term effects of 1,25(OH)_2_D_3_ on the mRNA expression of the highest-responding genes and performed a detailed time course measuring every 15 min over a period of 180 min (Fig. 4 right). Interestingly, the genes showed prominent differences in their timing. The *ZFP36* gene was already significantly up-regulated 15 min after onset of stimulation with 1,25(OH)_2_D_3_ (Fig. 4A), while the genes *SFT2D1* (Fig. 4B), *SEPT3* (Fig. 4C) and *SP100* (Fig. 4D) started to respond only after 90, 150 and 105 min, respectively. For comparison, the *MPC1* gene was after 135 min stimulation with 1,25(OH)_2_D_3_ consistently up-regulated, i.e. some 45 min delayed compared to the *SFT2D1* gene within the same chromatin domain (Fig. S8).

Taken together, every investigated chromatin domain contains at least one primary 1,25(OH)_2_D_3_ target gene, but these genes vary largely in the slope of their mRNA increase. Independent of their basal expression level, a few but not all neighboring genes respond delayed to stimulation with 1,25(OH)_2_D_3_, when compared to the master gene.

## Discussion

In this study, we investigated genomic binding sites of the transcription factor VDR in relation to the 3-dimensional organization of the human genome via chromatin domains. Such higher-order genome structures contribute to many nuclear functions, including the control of gene expression [43]. More than 90% of the 120,000 chromatin domains in K562 cells [33] contain multiple genes, i.e. transcription factors within these loops have the potential to activate more than one gene [44]. The high conservation of CTCF binding sites allows a reliable extrapolation of the CTCF ChIA-PET data from K562 cells to THP-1 cells. This means that for a general overview on the 3-dimensional chromatin organization in THP-1 cells the K562 ChIA-PET data are sufficient, but that for more detailed views the assay needs to be repeated in THP-1 cells. The number of chromatin domains largely exceeds the count of genome-wide VDR binding sites. Therefore, only a minority of these chromatin domains contains VDR sites. Those genes, which are co-located with one or more VDR binding sites found within the same chromatin domain, represent the group of possible primary targets of 1,25(OH)_2_D_3_. The chromatin domains around the genes *ZFP36*, *SFT2D1*, *SEPT3* and *SP100* are representative examples of small, mid-sized and large domains ranging from 17 to 590 kb and containing 3 to 7 genes.

The chromatin domains of the genes *ZFP36* and *SFT2D1* contain each three VDR binding sites. However, we found that, in both cases, one of the three VDR sites was dominant. These master VDR binding sites have very similar properties as those in the here examined domains that contain only one receptor locus, such as those of the genes *SEPT3* and *SP100*. Their association with VDR is ligand-inducible, as indicated by ChIP-seq data and confirmed by ChIP-qPCR time course experiments. Moreover, the local chromatin at these sites further opens after stimulation with 1,25(OH)_2_D_3_, as monitored by FAIRE-seq time course assays. In addition, the sites carry a DR3-type sequence below their VDR peak summits. Although DR3-type sequences are known since more than 20 years to be the preferred binding sites for VDR [12], [13], the recent VDR ChIP-seq datasets [7], [25]–[27] were consistent in reporting only for the minority of VDR peaks DR3-type motifs below their summits. This means that the presence of DR3-type sequences at VDR loci is rather an exception than the rule. Thus, the occurrence of master VDR sites is even more rare (160 sites in THP-1 cells). In the presence of a DR3-type binding site VDR recognizes genomic DNA as a heterodimer with retinoid X receptor [13], while in the absence of such a sequence the receptor may use a different heterodimerization partner or even may contact DNA indirectly via another transcription factor [23]. Accordingly, at genomic master loci VDR prefers to contact DNA as a “classical” heterodimer with retinoid X receptor.

VDR binding to its genomic loci is saturated after some 2 h. A comparable dynamic upload of a transcription factor to its binding sites has been shown before for RARγ [45] and C/EBPα and β [46]. Interestingly, the master VDR binding sites of the investigated primary 1,25(OH)_2_D_3_ target genes are indistinguishable in their time-dependent fashion of associating with VDR. This is in accordance with our previous comparison of six other master VDR binding sites, where we used a shorter time-scale for the ligand treatments [35]. Therefore, for periods longer than 2 h, the time of ligand stimulation in a VDR ChIP-seq experiment is not very critical, so that the different VDR ChIP-seq datasets should be comparable despite an individual ligand treatment protocol. In fact, the master VDR binding sites of the genes *ZFP36* and *SFT2D1* are also occupied in the two lymphoblastoid cell lines. Moreover, the VDR site of the *SP100* gene is even found in all published VDR ChIP-seq datasets [7], [25]–[27], i.e. only the VDR locus of the *SEPT3* gene seems to be specific to THP-1 cells. This suggests that master VDR binding sites may more likely be conserved between tissues and cell types than other VDR loci.

In all four example cases, the TSS region closest to the master VDR binding site turned out to be that of the fastest responding and most up-regulated primary 1,25(OH)_2_D_3_ target gene within the investigated chromatin domain. The distances range from 12 kb upstream to 8.5 kb downstream of the respective TSS. This would have been considered fairly large in the pre-genome era [47], but in fact they are rather small compared to the size of the respective chromosomal domain. However, this implies that the distance is still an important parameter, when searching for the most likely primary target gene of a master VDR binding site. Nevertheless, we found within each investigated chromatin loop at least one additional target of 1,25(OH)_2_D_3_. The kinetics of the up-regulation of the *MPC1* gene within the chromatin domain around the *SFT2D1* gene suggests that it is also a primary target of 1,25(OH)_2_D_3_, but it responds delayed compared to the master target gene. In contrast, the transient up-regulation (6 h after onset of stimulation with 1,25(OH)_2_D_3_) of the *PLEKHG2* gene within the *ZFP36* domain or the late responses (after 24 h) of the genes *NDUFA6* and *SP140L* within the domain of *SEPT3* and *SP100*, respectively, indicate that they are most likely secondary targets. For the overall physiological impact of vitamin D, a distinction between primary and secondary targets is less critical. However, for effective preventive intervention of healthy persons with vitamin D, or even a therapeutic application of 1,25(OH)_2_D_3_ analogs in the disease case, the mechanism and timing of responses to VDR ligands are important.

The genes *ZFP36*, *SFT2D1*, *MPC1*, *SEPT3* and *SP100* have not yet been explicitly described as primary targets of 1,25(OH)_2_D_3_. The protein product of *ZFP36* gene is the RNA binding protein tristetraprolin [48], which is involved in the negative control of cytokine gene expression [49]. The *SFT2D1* gene encodes for an uncharacterized vesicle transport protein, while the product of its neighbor *MPC1* is a mitochondrial pyruvate carrier protein [50]. The *SEPT3* gene encodes for a member of the septin family of GTPases, which are required for cytokinesis [51], and the SP100 protein is a nuclear body component [52]. Taken together, these new 1,25(OH)_2_D_3_ targets represent rather different physiological functions ranging from the control of immune response and metabolism to cellular growth. This re-emphasizes the pleiotropic function of vitamin D and its impact on cells of the hematopoietic system.

In conclusion, the observation of ligand-inducible VDR binding and chromatin opening in combination with a DR3-type sequence at the respective site is a very strong indication for an important VDR location with a high likelihood for at least one primary 1,25(OH)_2_D_3_ target gene within the same chromatin loop. This approach improves the identification and characterization of primary 1,25(OH)_2_D_3_ target genes and demonstrates the wide physiological impact of vitamin D.

## Supporting Information

Figure S1 **Chromatin domains determined by CTCF binding sites.** The IGV browser was used to display for the chromatin domains around the genes *ZFP36* (A), *SFT2D1* (B), *SEPT3* (C) and *SP100* (D) CTCF ChIP-seq data from the ENCODE cell lines K562 (red) and MCF-7 (blue) [33] and CTCF ChIA-PET data from K562 (light red) and MCF-7 (light blue) cells [33] in the track view (dark blue). Horizontal red lines indicate the core chromatin domains (as indicated in Table S3). The area of the genomic regions is identical to those shown in Fig. 1. Gene structures are shown in blue.
(PDF)

Figure S2 **Size range of VDR containing chromatin domains.** The distribution of the 1,599 VDR containing chromatin domains (Table S3) is shown for 14 size groups ranging from below 1 kb to 21 MB. The total number of domains per group is indicated in blue and the sub-group of those containing a master VDR binding site in red. The genes *ZFP36*, *SFT2D1*, *SEPT3* and *SP100* represent some the major size groups as indicated.
(PDF)

Figure S3 **Genomic view of VDR association and open chromatin at peaks 1 and 2 of the** ***SFT2D1*** **locus.** The IGV browser was used to visualize the genomic region of P1*_SFT2D1_* and P2*_SFT2D1_* (+/−15 kb of the center between both peaks). The peak tracks display data from a VDR ChIP-seq experiment in THP-1 cells (red, from unstimulated cells and after 40 min 1,25(OH)_2_D_3_ (1,25D) treatment [7]) and a time course of FAIRE-seq data from THP-1 cells (grey for EtOH-treated controls and turquoise for 1,25(OH)_2_D_3_ treatments for the indicated time periods [35]). Gene structures are shown in blue and VDR peak regions are shaded in grey.
(PDF)

Figure S4 **Definition of master VDR binding sites.** Within the list of 2,340 VDR peaks [7] 627 show an enhancement of at least 9-fold (red), 709 have a FAIRE signal that is at least 1.1-fold induced (green) and 739 carry a DR3-type sequence with a HOMER score of at least 7 (blue). The center of the Venn diagram indicates 160 VDR peaks that share all three properties and are therefore considered as master VDR loci.
(PDF)

Figure S5 **Dynamics of VDR association.** ChIP-qPCR was performed to determine VDR association (blue) and unspecific IgG binding (grey) at P1*_ZFP36_* (A), P2*_ZFP36_* (B), P1*_SFT2D1_* (C) and P2*_SFT2D1_* (D) and the negative control region of the *MB* gene (E). THP-1 cells were stimulated for 1, 2, 3, 4, 5 and 24 h with 100 nM 1,25(OH)_2_D_3_ and chromatin was extracted. The data points represent the means of at least three independent experiments and the bars indicate standard deviations. The unspecific background binding at the negative control region (E) was subtracted from A–D. Two-tailed Student’s t-tests were performed to determine the significance of VDR association in reference to IgG background (*p<0.05; **p<0.01).
(PDF)

Figure S6 **Basal mRNA expression of the genes within the four exemplary chromatin domains.** qPCR was performed to determine the relative basal expression of all genes within the chromatin loop used in this study (normalized to the reference genes *B2M*, *GAPDH* and *HPRT1*) in untreated THP-1 cells. The data points represent the means of three independent experiments (each performed in triplicate) and the bars indicate standard deviations.
(PDF)

Figure S7 **Time course expression profiling of neighboring genes.** qPCR was performed to determine the relative changes of mRNA expression of the genes that co-locate with the VDR target genes *ZFP36* (A), *SFT2D1* (B), *SEPT3* (C) and *SP100* (D) in the same chromatin loop normalized by the three reference genes *B2M*, *GAPDH* and *HPRT1*. THP-1 cells were incubated with 100 nM 1,25(OH)_2_D_3_ for 2, 4, 6 and 24 h. The columns represent the means of three independent experiments (each performed in triplicate) and the bars indicate standard deviations. Two-tailed Student’s t-tests were performed to determine the significance of the mRNA induction by 1,25(OH)_2_D_3_ in reference to solvent-treated cells (*p<0.05; **p<0.01; ***p<0.001).
(PDF)

Figure S8 **Detailed time course expression profiling of the** ***MPC1*** **gene.** qPCR was performed to determine the relative changes of mRNA expression of the *MPC1* gene normalized by the three reference genes *B2M*, *GAPDH* and *HPRT1*. THP-1 cells were incubated at 15 min intervals over a time period of 180 min. Data points represent the means of three independent experiments (each performed in triplicate) and the bars indicate standard deviations. Two-tailed Student’s t-tests were performed to determine the significance of the mRNA induction by 1,25(OH)_2_D_3_ in reference to solvent-treated cells (*p<0.05; **p<0.01).
(PDF)

Table S1 **ChIP-qPCR primers.**
(PDF)

Table S2 **Reverse transcription qPCR primers.**
(PDF)

Table S3 **VDR ChIP-seq peaks associated with chromatin loops.** The 2,340 VDR ChIP-seq peaks found in THP-1 cells [7] were aligned with the closest CTCF peaks (obtained from ChIA-PET data from K562 cells [33]) left and right of them. The distance between the summits of both CTCF peaks determines the size of the respective core chromatin loop. In addition, the number of VDR peaks in the same loop, averages of 1,25(OH)_2_D_3_ (1,25D)- and EtOH-treated FAIRE-seq signals [35], their fold change and the significance of this ligand effect as well as the sequence and position of DR3-type sequences below the VDR peak summits [7] are listed. A previous study [35] had indicated that a number of the VDR ChIP-seq peaks are likely to be artifacts. An example for this is the first VDR site of the chromatin loop around the *ZFP36* gene, which therefore was not investigated in this study.
(XLSX)

Table S4 **Primary 1,25(OH)_2_D_3_ target genes are enriched with VDR containing chromatin domains.** The 408 significantly up-regulated primary 1,25(OH)_2_D_3_ target genes in THP-1 cells [7] were classified by their location within one of the 1,599 VDR containing chromatin domains or even within those containing a master VDR site. For further evaluations, the distance to the closest VDR domain and the closest VDR master domain are indicated.
(XLSX)
