# Supplementary figures and images for: Characterization of Genomic Vitamin D Receptor Binding Sites through Chromatin Looping and Opening
Source: PLoS One. 2014 Apr 24;9(4):e96184. doi: 10.1371/journal.pone.0096184 (PMC3999108; doi:10.1371/journal.pone.0096184)

**Fig. S1**

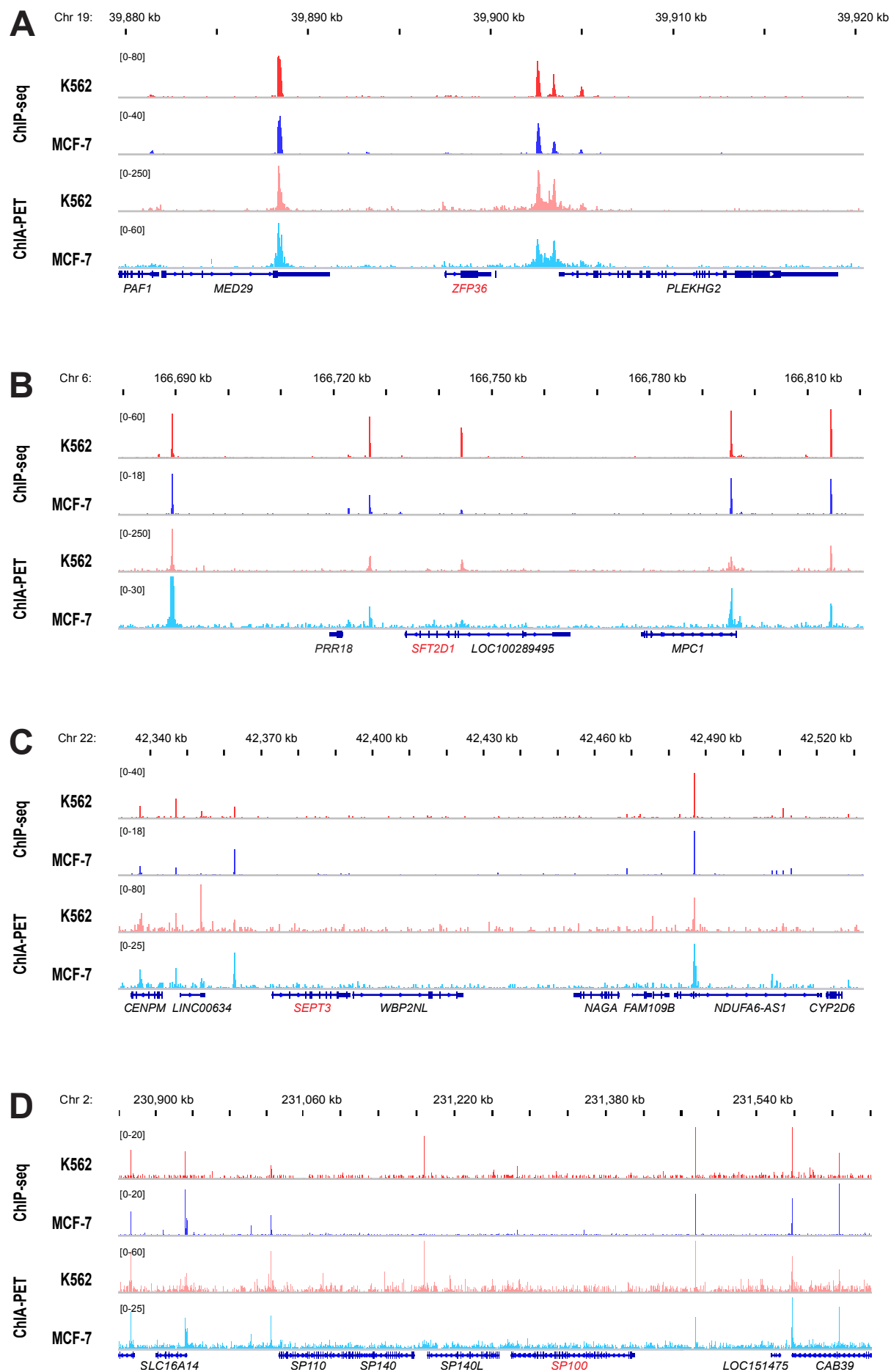

Supplement: Figure S1 — Chromatin domains determined by CTCF binding sites. The IGV browser was used to display for the chromatin domains around the genes ZFP36 (A), SFT2D1 (B), SEPT3 (C) and SP100 (D) CTCF ChIP-seq data from the ENCODE cell lines K562 (red) and MCF-7 (blue) [33] and CTCF ChIA-PET data from K562 (light red) and MCF-7 (light blue) cells [33] in the track view (dark blue). Horizontal red lines indicate the core chromatin domains (as indicated in Table S3). The area of the genomic regions is identical to those shown in Fig. 1. Gene structures are shown in blue. (PDF) [file pone.0096184.s001.pdf]

Fig. S2

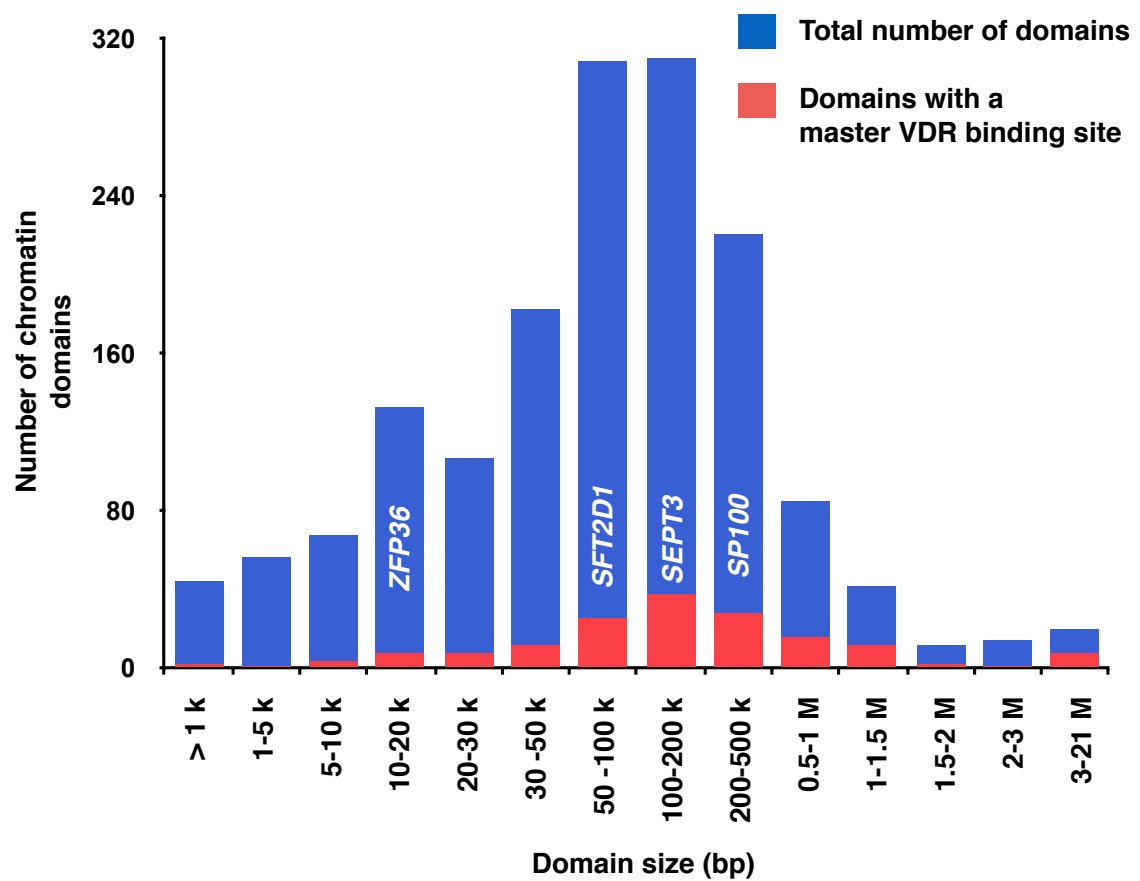

Supplement: Figure S2 — Size range of VDR containing chromatin domains. The distribution of the 1,599 VDR containing chromatin domains (Table S3) is shown for 14 size groups ranging from below 1 kb to 21 MB. The total number of domains per group is indicated in blue and the sub-group of those containing a master VDR binding site in red. The genes ZFP36, SFT2D1, SEPT3 and SP100 represent some the major size groups as indicated. (PDF) [file pone.0096184.s002.pdf]

Fig. S3

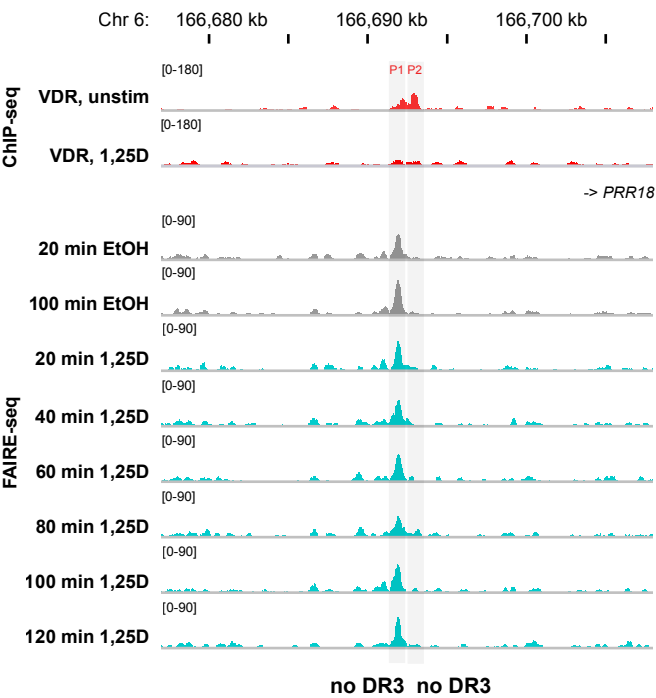

Supplement: Figure S3 — Genomic view of VDR association and open chromatin at peaks 1 and 2 of the SFT2D1 locus. The IGV browser was used to visualize the genomic region of P1SFT2D1 and P2SFT2D1 (+/−15 kb of the center between both peaks). The peak tracks display data from a VDR ChIP-seq experiment in THP-1 cells (red, from unstimulated cells and after 40 min 1,25(OH)2D3 (1,25D) treatment [7]) and a time course of FAIRE-seq data from THP-1 cells (grey for EtOH-treated controls and turquoise for 1,25(OH)2D3 treatments for the indicated time periods [35]). Gene structures are shown in blue and VDR peak regions are shaded in grey. (PDF) [file pone.0096184.s003.pdf]

**Fig. S4**

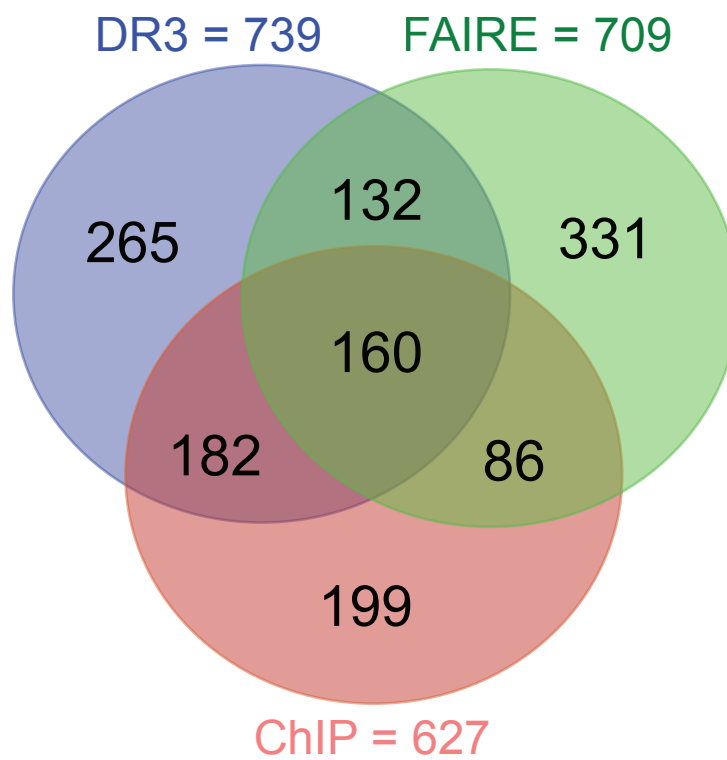

Supplement: Figure S4 — Definition of master VDR binding sites. Within the list of 2,340 VDR peaks [7] 627 show an enhancement of at least 9-fold (red), 709 have a FAIRE signal that is at least 1.1-fold induced (green) and 739 carry a DR3-type sequence with a HOMER score of at least 7 (blue). The center of the Venn diagram indicates 160 VDR peaks that share all three properties and are therefore considered as master VDR loci. (PDF) [file pone.0096184.s004.pdf]

Fig. S5

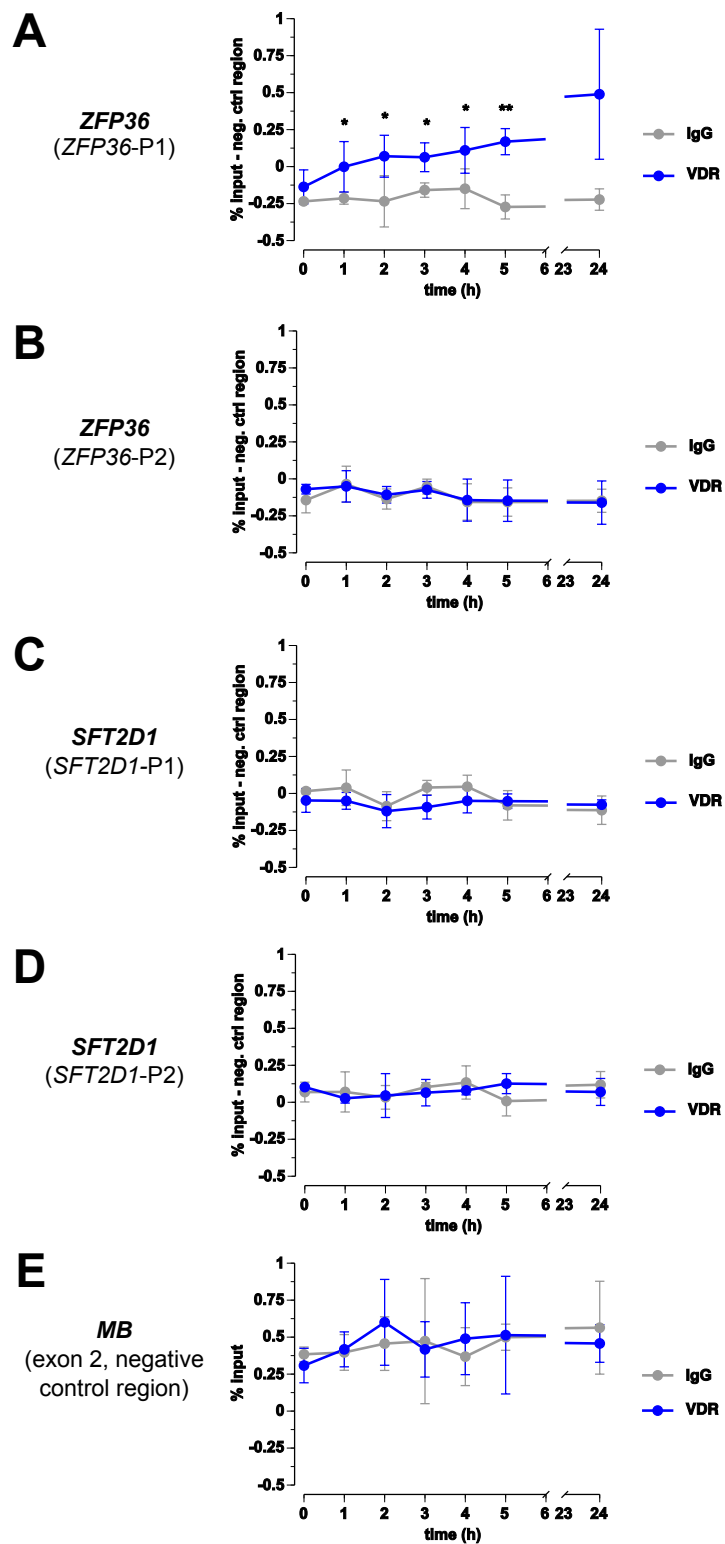

Supplement: Figure S5 — Dynamics of VDR association. ChIP-qPCR was performed to determine VDR association (blue) and unspecific IgG binding (grey) at P1ZFP36 (A), P2ZFP36 (B), P1SFT2D1 (C) and P2SFT2D1 (D) and the negative control region of the MB gene (E). THP-1 cells were stimulated for 1, 2, 3, 4, 5 and 24 h with 100 nM 1,25(OH)2D3 and chromatin was extracted. The data points represent the means of at least three independent experiments and the bars indicate standard deviations. The unspecific background binding at the negative control region (E) was subtracted from A–D. Two-tailed Student’s t-tests were performed to determine the significance of VDR association in reference to IgG background (*p<0.05; **p<0.01). (PDF) [file pone.0096184.s005.pdf]

Fig. S6

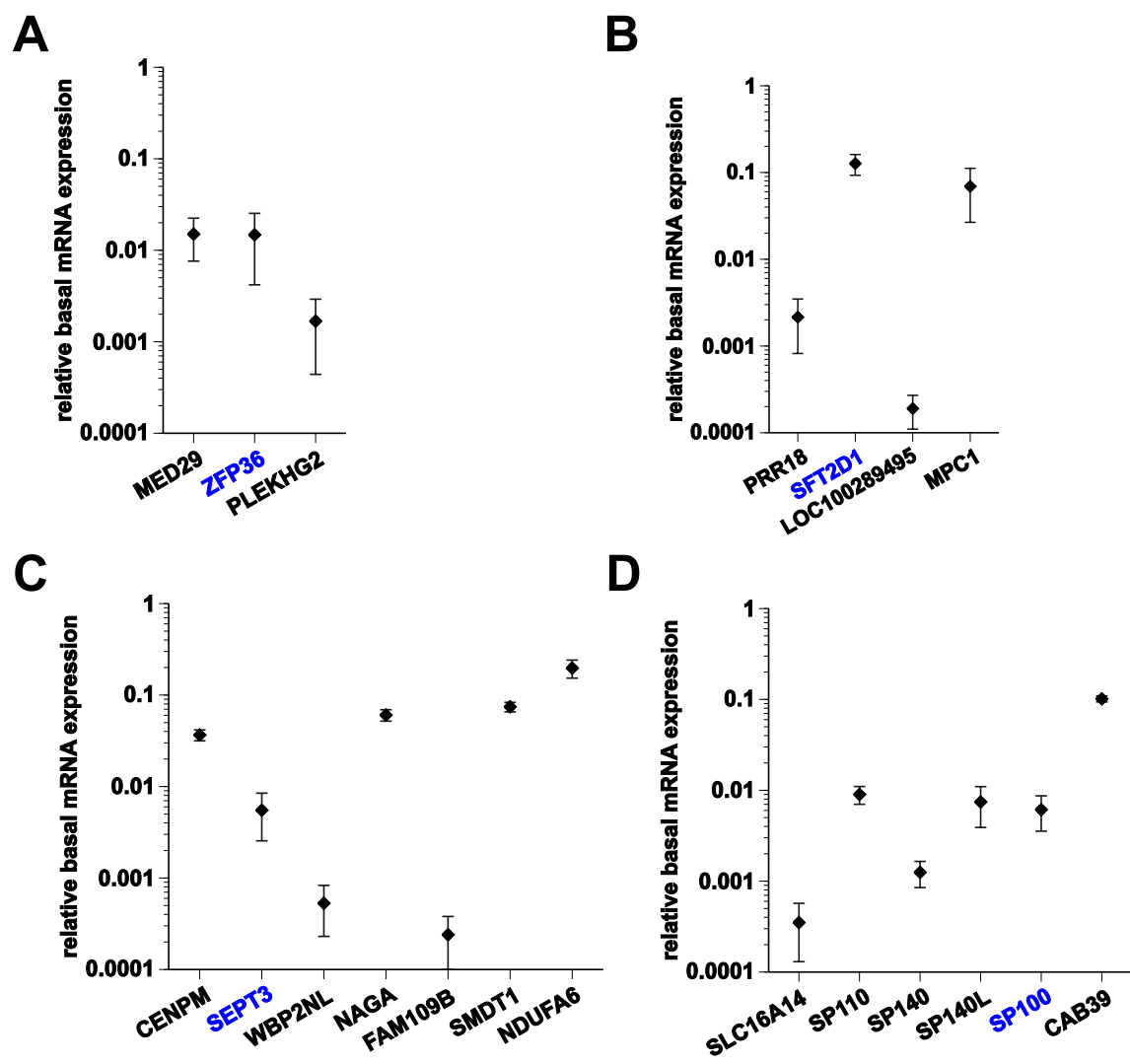

Supplement: Figure S6 — Basal mRNA expression of the genes within the four exemplary chromatin domains. qPCR was performed to determine the relative basal expression of all genes within the chromatin loop used in this study (normalized to the reference genes B2M, GAPDH and HPRT1) in untreated THP-1 cells. The data points represent the means of three independent experiments (each performed in triplicate) and the bars indicate standard deviations. (PDF) [file pone.0096184.s006.pdf]

Fig. S7

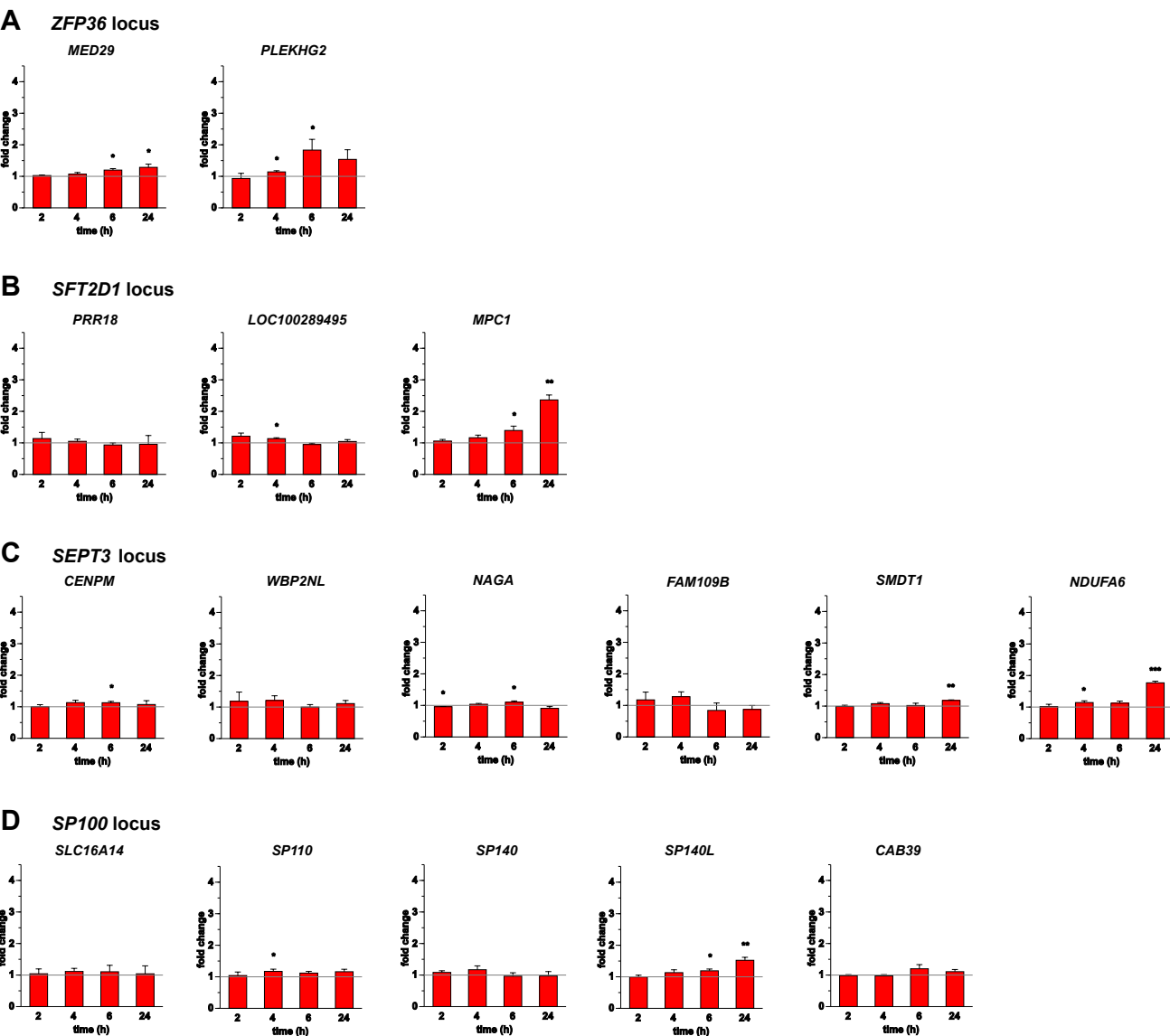

Supplement: Figure S7 — Time course expression profiling of neighboring genes. qPCR was performed to determine the relative changes of mRNA expression of the genes that co-locate with the VDR target genes ZFP36 (A), SFT2D1 (B), SEPT3 (C) and SP100 (D) in the same chromatin loop normalized by the three reference genes B2M, GAPDH and HPRT1. THP-1 cells were incubated with 100 nM 1,25(OH)2D3 for 2, 4, 6 and 24 h. The columns represent the means of three independent experiments (each performed in triplicate) and the bars indicate standard deviations. Two-tailed Student’s t-tests were performed to determine the significance of the mRNA induction by 1,25(OH)2D3 in reference to solvent-treated cells (*p<0.05; **p<0.01; ***p<0.001). (PDF) [file pone.0096184.s007.pdf]

**Fig. S8**

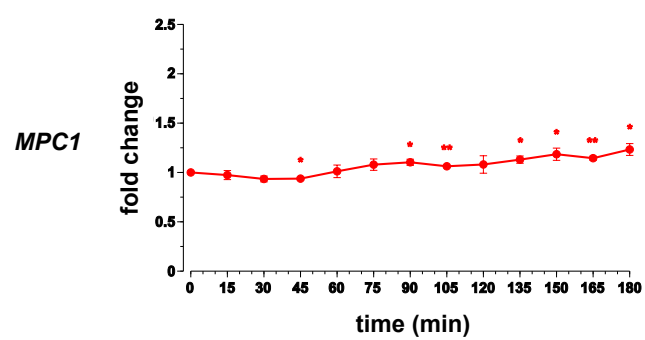

Supplement: Figure S8 — Detailed time course expression profiling of the MPC1 gene. qPCR was performed to determine the relative changes of mRNA expression of the MPC1 gene normalized by the three reference genes B2M, GAPDH and HPRT1. THP-1 cells were incubated at 15 min intervals over a time period of 180 min. Data points represent the means of three independent experiments (each performed in triplicate) and the bars indicate standard deviations. Two-tailed Student’s t-tests were performed to determine the significance of the mRNA induction by 1,25(OH)2D3 in reference to solvent-treated cells (*p<0.05; **p<0.01). (PDF) [file pone.0096184.s008.pdf]
