# Supplementary material for: Characterization of Genomic Vitamin D Receptor Binding Sites through Chromatin Looping and Opening
Source: PLoS One. 2014 Apr 24;9(4):e96184. doi: 10.1371/journal.pone.0096184 (PMC3999108; doi:10.1371/journal.pone.0096184)
Supplement: Table S1 — ChIP-qPCR primers. (PDF) [file pone.0096184.s009.pdf]

Genomic region	Fragment size (bp)	Annealing temperature (°C)	Primer sequences (5'-3')
MB[1]	76	60	AAGTTTGACAAGTTCAAGCACCTG
exon 2			TGGCACCATGCTTCTTTAAGTC
SEPT3[2]	104	65	GAAGGCTTAGACCCAGACTGAGAAC
P_{SEPT3}			ATTAGCATTGTAATGGGGAAAGCTG
SFT2D1[2]	153	65	AAATTGTTTCCCAAAGCATCTGCACTA
$P1_{SFT2D1}$			TTGACCACAACACGATATCACTTCACA
SFT2D1[2]	90	65	TGTGCTCTCCTCCCACACAACTAGA
$P2_{SFT2D1}$			CCGTGATTTGGCAGCTTAGTAATGG
SFT2D1[2]	125	65	CCCCACACTTCCATTCTTAGCAGAC
$P3_{SFT2D1}$			TAGTTGGTGTGTTTGGTGGTTTGGT
SP100[2]	95	65	AGCTGACCGGGACACTCTAA
P_{SP100}			GAGGAAGGCTGAGGGGTGAA
ZFP36[2]	139	65	CCTTTAAGCTGCGTAAGGATTTGGA
$P1_{ZFP36}$			ACACTCGGGTTCCTCTCCTGTAACC
ZFP36[2]	148	65	ACCCCAAATACAAGACGGAACTCTG
$P2_{ZFP36}$			GAGAAGCTGATGCTCTGGCGAAG
ZFP36[2]	141	65	AGACCATACAAGGCCAGCGTCGTC
$P3_{ZFP36}$			GAGTGCGGGGCAAGGAGAAAAA

[1] negative control (Red ChIP Kit (Diagenode))

[2] designed using Primer3 (http://frodo.wi.mit.edu)
