# Supplementary material for: Characterization of Genomic Vitamin D Receptor Binding Sites through Chromatin Looping and Opening
Source: PLoS One. 2014 Apr 24;9(4):e96184. doi: 10.1371/journal.pone.0096184 (PMC3999108; doi:10.1371/journal.pone.0096184)
Supplement: Table S2 — Reverse transcription qPCR primers. (PDF) [file pone.0096184.s010.pdf]

Gene	Fragment size (bp)	Annealing temperature (°C)	Primer sequences (5'-3')
B2M[1,2]	246	60	GGCTATCCAGCGTACTCCAAA
			CGGCAGGCATACTCATCTTTTT
CAB39[2]	88	60	TGAACCTGCTGCGAGACAAAA
			TGCGTCTTGTTAGGATTGGCTA
CENPM[2]	146	64	GCGGACTCGATGCTCAAAGA
			TTCTGGAGACTGTATTTGCTGTG
FAM109B[5]	137	65	TCAAGGGCAACCTGCTATTC
			ATCAAAGCAGATGGCAAAGG
GAPDH[1,2]	113	60	CATGAGAAGTATGACAACAGCCTA
			GTCCTTCCACGATACCAAAGT
HPRT1[1,3]	94	60	TGACACTGGCAAAACAATGCA
			GGTCCTTTTCACCAGCAAGCT
LOC100289495[4]	196	64	TTTCTCTCGGACCCGGTAGT
			AAGTGCAGGGTTCAGTGGTC
MED29[2]	125	60	CTTCGATCCTGTGCAGCGTTA
			TGCTCTTTTGTCCATTGTCGATG
MPC1[2]	47	60	AGTCTCCAGAGATTATCAGTGGG
			GCAACAGAGGGCAAATGTCAT
NAGA[6]	220	58	CTGAACTTCACCGGGTCTGT
			GCTCCATGGTCTAGGCTCAG
NDUFA6[2]	152	65	GACGGGATAAAGTCCGAGAAATG
			TTCATGGAAGAACCGCATAACA
PLEKHG2[2]	203	60	AGACAGAACGGGCCTATGTCA
			GAAGCACTCGGCAATACCC
PRR18[4]	86	64	GAGCATCCATGCAGTTCGTG
			TCTGGGACCTAGACGAACCC
SEPT3[2]	105	65	CGCTGGTCAACACGCTCTT
			CATGCCCGATAGCTTTGATCT
SFT2D1[2]	127	60	GCCATCTGCTTCGTATGTGG
			ATGTACTGGCTAACGCAGCAA
SLC16A14[2]	118	65	GTGTCCTCAACGTGGAATGG
			ATGAACAAGCCGATGAAAGGG
SMDT1[2]	75	65	ACCGTCGAGGTCAGTCATC
			AGAAGGCCGAAGGACATTTTC
SP100[2]	118	60	AAGCCAACACTAGACCTTTGAAA
			GTGCCCTTCACCTCACCAC
SP110[2]	191	60	CAGCACACCTTCAGACAAGAA
			TCTCTACCGTGGAGTTACAAGTT
SP140[2]	123	65	AGGATGGTCGCAGAGATCCA
			TGGCCTTGTTATTGCACTTGC
SP140L[2]	123	60	GGGCTGAACGGAGGTGTTT
			AGTGTCATAGACAAGTCCCTCAT
WBP2NL[2]	119	60	CGGTGAAAGTCTCTTGAAGCG
			CCCGGTATGAAGTGAGAAACAA
ZFP36[5]	104	65	CTCATGGCCAACCGTTACAC
			GACTCAGTCCCTCCATGGTC

[1] reference gene
[2] Sequence obtained from PrimerBank (http://pga.mgh.harvard.edu/primerbank)
[3] see [39]
[4] designed with Primer-BLAST (http://www.ncbi.nlm.nih.gov/tools/primer-blast)
[5] designed with Primer3Plus (http://www.bioinformatics.nl/cgi-bin/primer3plus/primer3plus.cgi)
